# 296. Description of Super-infections in Hospitalized Patients with COVID-19

**DOI:** 10.1093/ofid/ofab466.498

**Published:** 2021-12-04

**Authors:** Geena Kludjian, Stephanie Spivack, Stefania Gallucci, Laurie Kilpatrick, Aaron D Mishkin, Umadevi Sajjan, Vincent Tam, Jason C Gallagher, Jason C Gallagher

**Affiliations:** 1 Temple University School of Pharmacy, Philadelphia, Pennsylvania; 2 Temple University Hospital, Philadelphia, Pennsylvania; 3 Temple University, Philadelphia, Pennsylvania; 4 Temple University School of Medicine, Philadelphia, Pennsylvania; 5 Lewis Katz School of Medicine at Temple University, Philadelphia, Pennsylvania

## Abstract

**Background:**

The rate of bacterial and fungal super-infections (SI) in inpatients with COVID-19 is unknown. In this study, we aimed to identify and describe patients that developed secondary infections while hospitalized with COVID-19.

**Methods:**

We performed a retrospective chart review on patients admitted to our health system between March and May 2020 with confirmed COVID-19 by nasopharyngeal PCR. We reviewed patients with positive cultures from urine, blood, sputum, and sterile sites. Patients with positive cultures had cases reviewed to determine if they represented a true infection, defined by CDC criteria. SIs were defined as infections that occurred at least 48 hours or longer after the initial positive SARS-CoV-2 test. Additional data was collected on patient demographics, COVID-related therapies, types of infections, and outcomes.

**Results:**

902 patients were admitted with COVID-19 during our study period. Of these, 52 patients (5.8%) developed a total of 82 SIs. Tables 1 and 2 describe patient and infection characteristics. Patients identified as having a SI were admitted for a median of 30 days; 56% had mortality, and 39% of remaining patients were readmitted within 90 days.

Table 1. Patient Characteristics

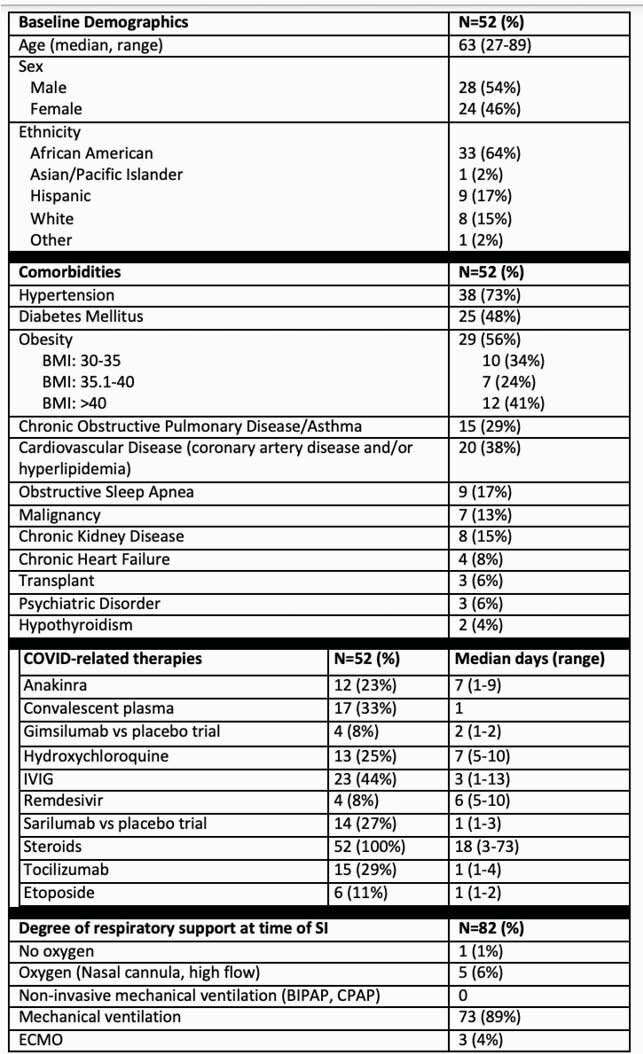

Table 2. Super-infections

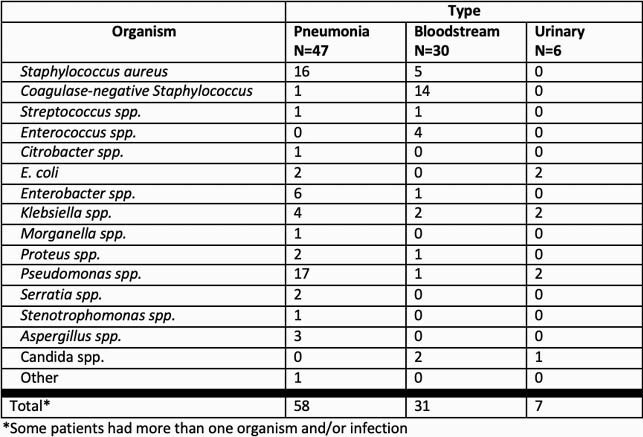

**Conclusion:**

Overall, the rate of SIs in patients admitted with COVID-19 is low. These patients had a long length of stay, which may be either a cause of SI or an effect. Further analysis with matched COVID-positive control patients who do not develop SIs is needed to evaluate the risk of development of SIs in relation to presenting respiratory status, COVID-related therapies, and other patient-specific factors.

**Disclosures:**

**Jason C. Gallagher, PharmD, FIDP, FCCP, FIDSA, BCPS**, **Astellas** (Consultant, Speaker’s Bureau)**Merck** (Consultant, Grant/Research Support, Speaker’s Bureau)**Qpex** (Consultant)**scPharmaceuticals** (Consultant)**Shionogi** (Consultant) **Jason C. Gallagher, PharmD, FIDP, FCCP, FIDSA, BCPS**, Astellas (Individual(s) Involved: Self): Speakers’ bureau; Merck (Individual(s) Involved: Self): Consultant, Grant/Research Support; Nabriva: Consultant; Qpex (Individual(s) Involved: Self): Consultant; Shionogi (Individual(s) Involved: Self): Consultant

